# High HAM-A-Detected Prevalence of Anxiety in Parkinson’s Disease, but No Correlation with Severity of Motor Disease

**DOI:** 10.3390/diseases14090338

**Published:** 2026-09-15

**Authors:** Khaoula Elcadi, Oussama Cherkaoui Rhazouani, Nissrine Louhab, Najib Kissani, Mohamed Chraa

**Affiliations:** 1 Clinical Experimental and Environmental Neuroscience Laboratory, Faculty of Medicine and Pharmacy of Marrakech, Cadi Ayaad University, Marrakech 40000, Morocco; 2 Neurology Department, Military Hospital Avicenne, Marrakech 40000, Morocco; 3 Neuroscience Research Laboratory, Marrakech Medical School, Cadi Ayyad University, Marrakech 40000, Morocco; 4 Department of Neurology, Mohammed VI University Medical Center, Marrakesh 40000, Moroccom.chraa@uca.ma (M.C.)

**Keywords:** Parkinson’s disease, anxiety, motor severity, Hamilton Anxiety Rating Scale (HAM-A)

## Abstract

Background: In Parkinson’s disease (PD), non-motor symptoms especially anxiety represent a significant and underappreciated burden. Despite the lack of particular validation in this demographic, the Hamilton Anxiety Rating Scale (HAM-A) is widely used in clinical and research contexts. Methods: A cross-sectional study of 200 Parkinson’s disease patients (mean age 62.77 ± 8.32 years; 118 men and 82 females) was conducted. The Hoehn & Yahr (H&Y) scale was used to grade motor severity, whereas the HAM-A was used to measure anxiety. Results: Of the patients, 52.5% of people (*n* = 105; mean score 13.85 ± 6.09) had anxiety (HAM-A ≥ 14). Age (r = 0.032; *p* = 0.657), disease duration (r = 0.115; *p* = 0.105), H&Y stage (r = −0.073; *p* = 0.305), and sex (*p* = 0.856) did not significantly correlate with the HAM-A score. Conclusion: Although the HAM-A shows a high incidence of anxiety in Parkinson’s disease (PD), its consistent lack of connection with clinical illness markers raises serious concerns about construct validity, which are probably caused by somatic item overlap. It is best to use disease-specific tools like the GAD-7 or Parkinson Anxiety Scale (PAS).

## 1. Introduction

Parkinson’s disease (PD), which affects about 1% of persons over 60 and an estimated 8.5 million people worldwide as of 2019 [1,2], is the second most common neurodegenerative ailment in the world. An increasing body of research currently shows that non-motor symptoms (NMS) form an essential and frequently dominant dimension of the disease burden, despite the fact that the clinical definition of Parkinson’s disease (PD) has historically focused on its cardinal motor features, resting tremor, bradykinesia, and stiffness [3,4].

Anxiety disorders are among the most common and incapacitating symptoms of NMS, although they are routinely underdiagnosed and undertreated in clinical practice [5].

Due to differences in patient demographics, diagnostic criteria, and the psychometric tools used, reported prevalence estimates range greatly, from 25% to 70% [6,7]. The significance of choosing assessment instruments with suitable validity for this particular demographic is highlighted by this heterogeneity.

Developed by Max Hamilton in 1959 [8], the Hamilton Anxiety Rating Scale (HAM-A) is still one of the most popular clinician-rated measures of anxiety severity in the world. It covers 14 items examining both psychological and bodily elements of anxiety, each rated from 0 (absent) to 4 (extremely severe). However, the somatic subscale items of the HAM-A, such as muscular tension, tremors, sleep disturbances, and autonomic symptoms, significantly overlap with intrinsic manifestations of Parkinson’s disease (PD), regardless of any comorbid anxiety state [9,10]. The HAM-A was initially developed and validated in a general psychiatric population. This possibility of semiological contamination could result in a systematic overestimation of anxiety severity and reduce the instrument’s discriminative validity in this situation.

Additionally, there are significant differences in the phenomenology of anxiety in PD and basic anxiety disorders. The distinctive phenomenology of anxiety in Parkinson’s disease (PD) is sometimes poorly captured by diagnostic criteria and generic scales intended for the general population. This specificity is demonstrated by three presentations: fluctuating anxiety associated with OFF-state dopaminergic medication phases, anticipatory anxiety linked to freezing of gait (anxiety itself can cause or exacerbate freezing episodes, and the most prevalent atypical presentation, fear of falling, which is frequently more common than classic generalized anxiety in Parkinson’s disease [11,12].

A fundamental underlying assumption of the clinical application of the HAM-A in Parkinson’s disease (PD) is that, if the scale accurately measures anxiety associated with the disease, its scores should correspond with indicators of PD severity and progression (e.g., disease duration, motor stage), in line with the established correlation between increasing motor impairment and psychological distress in PD. On the other hand, the scale’s discriminative validity in this cohort would be called into question if there was no such correlation, even though there seemed to be a high prevalence of elevated scores. This would suggest that the scale is measuring somatic burden associated with Parkinson’s disease rather than a unique anxiety construct.

The current study intends to (i) ascertain the prevalence of anxiety as determined by the HAM-A in a cohort of 200 patients with Parkinson’s disease (PD); (ii) investigate correlations, examining the hypothesis that the HAM-A’s scores should correspond with clinical variables, severity indicators such as age, sex, disease duration, and motor stage if it accurately measures anxiety associated to Parkinson’s disease; and (iii) discuss the methodological implications of these findings for clinical and research practice.

## 2. Participants and Study Design

This hospital based, cross-sectional observational study included 200 consecutive outpatients diagnosed with Parkinson’s disease (PD) according to the 2015 Movement Disorder Society (MDS) clinical diagnostic criteria [13].

Prerequisites for inclusion were being at least 40 years old, having a confirmed diagnosis of Parkinson’s disease, and being able to give informed consent.

Individuals with corticobasal syndrome, progressive supranuclear palsy, multiple system atrophy, severe psychiatric comorbidity that prevented a valid assessment, or substantial communication impairment, were not included. Each participant provided written informed permission prior to enrollment, and the institutional ethics committee approved the study procedure.

### 2.1. Clinical Evaluations

The degree of anxiety was assessed by a trained neuropsychologist using the validated French version of the Hamilton Anxiety Rating Scale (HAM-A). Each of the 14 items was rated from 0 (absence) to 4 (very severe/incapacitating), for a total score of 0–56. No anxiety (<14), moderate anxiety (14–17), and severe anxiety (≥18) were the three pre-established clinical severity criteria [8].

Patients were categorized from stage 1 (unilateral involvement only) to stage 5 (bedridden or wheelchair-bound) using the Hoehn & Yahr (H&Y) scale [14]. Clinical data (disease duration in years) and sociodemographic data (age, sex) were collected through structured interviews and a review of medical records.

### 2.2. Analysis of Statistics

Frequencies and percentages are used to express categorical data, whereas mean ± standard deviation (SD) is used to express continuous variables. The HAM-A scores by sex were compared between groups using the independent-samples Student’s *t*-test.

Pearson’s correlation coefficients (r) were computed to assess linear correlations between HAM-A scores and continuous clinical parameters (age, disease duration, and H&Y stage). HAM-A score differences across H&Y categories were evaluated using one-way analysis of variance (ANOVA). A two-tailed threshold of *p* < 0.05 was established for statistical significance.

## 3. Results

### 3.1. Clinical and Demographic Features

Out of the 200 patients in the study group, 118 were male (59%) and 82 were female (41%), resulting in a male-to-female ratio of 1.44:1. The average age was 62.77 ± 8.32 years (range: 43.7–83.8), and the average length of the illness was 3.42 ± 1.76 years (range: 0.5–9.3). The average Hoehn & Yahr stage was. 2.59 ± 0.91 (Table 1).

**Figure 1:**
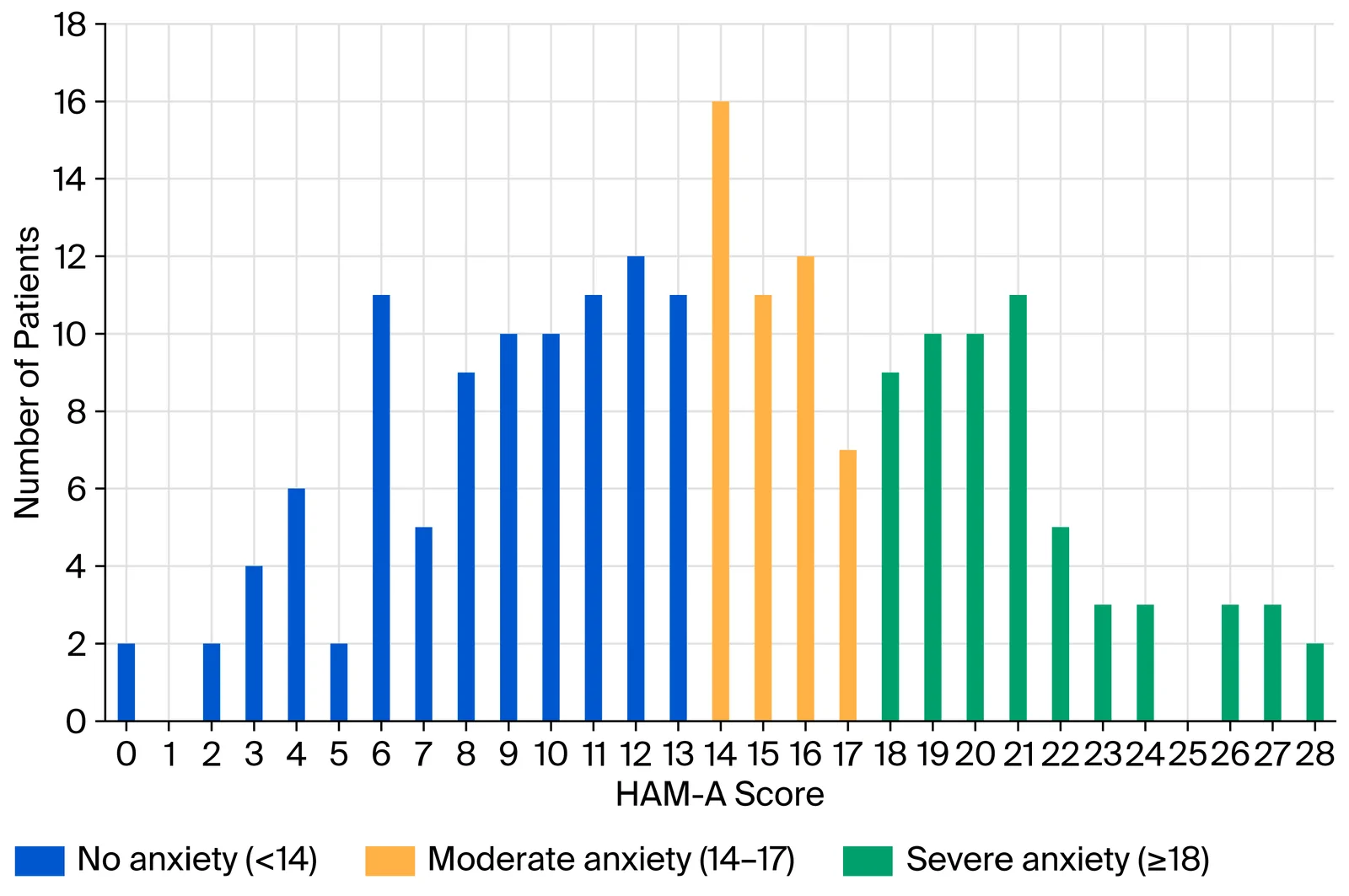
Distribution of HAM-A scores, *n* = 200.

**Figure 2:**
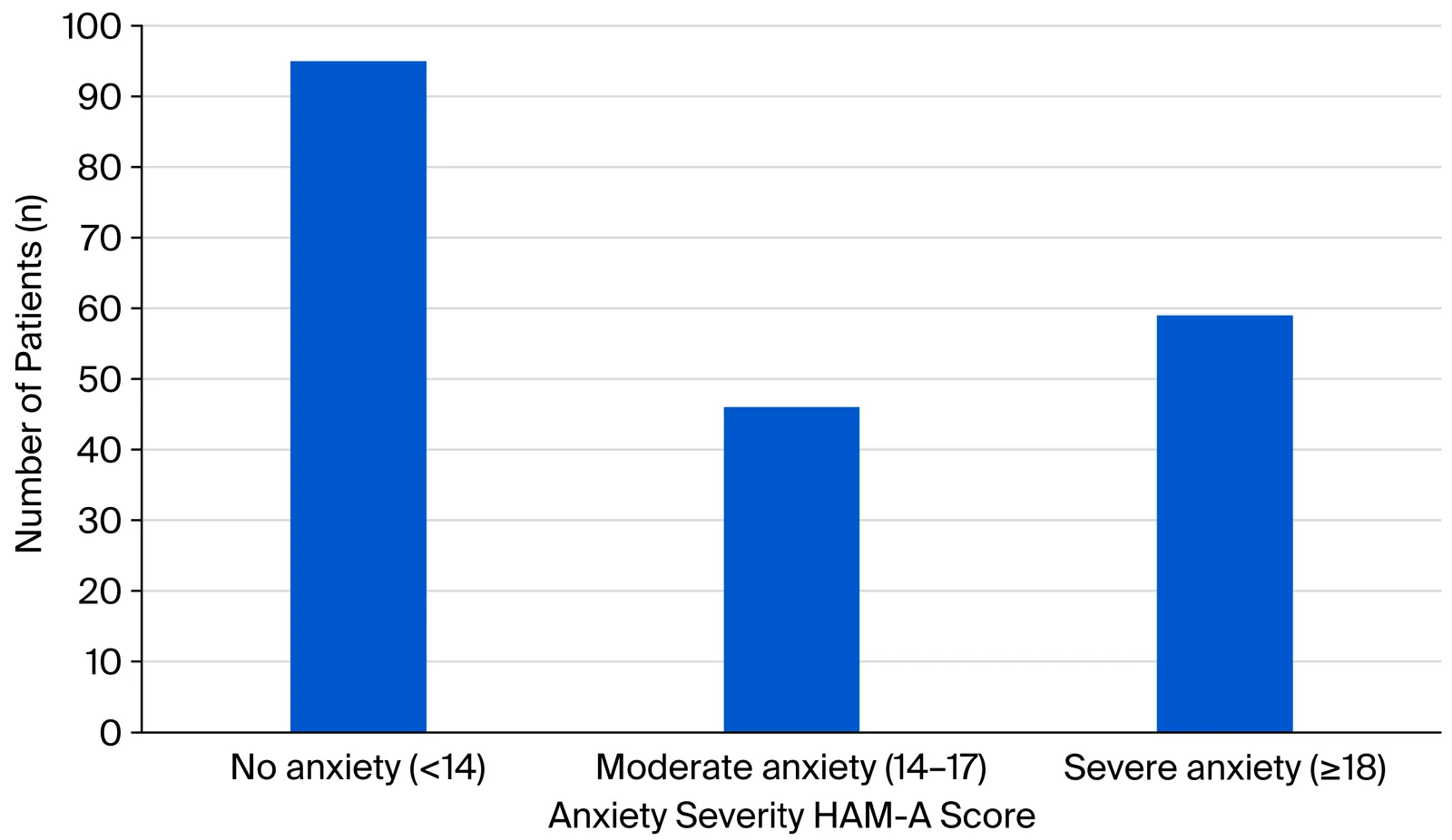
Prevalence of anxiety by HAM-A category, *n* = 200.

Stage I: Unilateral (*n* = 14, 7.0%), Stage II: Bilateral (*n* = 67, 33.5%), Stage III: Bilateral with postural instability (*n* = 89, 44.5%), Stage IV: Severe handicap (*n* = 13, 6.5%), and Stage V: Wheelchair (*n* = 17, 8.5%) (Figure 1 and Figure 2).

### 3.2. Anxiety Prevalence and Severity

Overall, 93.0% (*n* = 186/200) of people had clinically significant anxiety (HAM-A ≥ 5). Just 14 patients (7.0%) did not exhibit any anxiety that could be identified clinically. The HAM-A score ranged from 0 to 28, with a mean of 13.85 ± 6.09. The distribution of severity was as follows: 97 patients (48.5%) had mild anxiety (HAM-A 5–14), 70 patients (35.0%) had moderate anxiety (15–21), and 19 patients (9.5%) had severe anxiety (22–28). In total, moderate-to-severe anxiety was reported by 44.5% of the cohort.

### 3.3. Clinical Subgroup Analysis

Sex: The average HAM-A score was 13.92 ± 6.02 for men and 13.76 ± 6.22 for women. There was no statistically significant difference between the sexes, according to an independent-samples *t*-test(t = −0,181; *p* = 0.856). Age: There was no linear relationship between age and HAM-A score, as indicated by the Pearson’s correlation of r = 0.032 (*p* = 0.657, Table 2).

A small but non-significant tendency toward higher scores in older patients was revealed by age group analysis (40–55, 55–65, 65–75, and 75+) (mean scores: 12.95, 13.83, 14.32, and 14.63, respectively).

Disease length: A weakly positive but non-significant trend was revealed by Pearson’s correlation between disease duration and HAM-A scores, which was r = 0.115 (*p* = 0.105). Hoehn & Yahr stage: HAM-A scores did not significantly change between HY stages, according to a one-way ANOVA (F = 0.891; *p* = 0.621). There was no discernible monotonic trend in the mean scores, which varied from 11.92 (Stage IV) to 14.43 (Stage I).

## 4. Correlation Analyses

**Figure 3:**
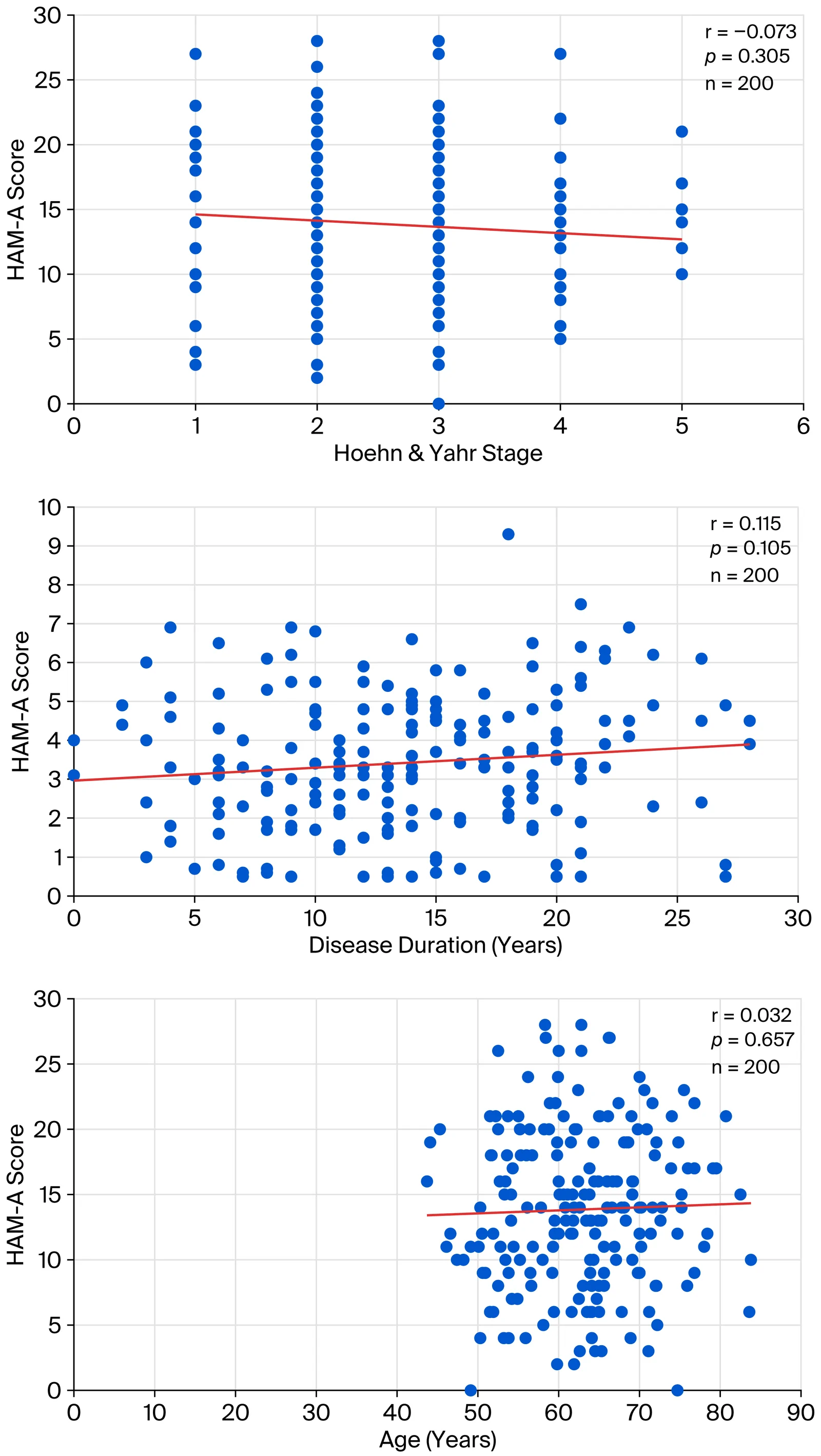
Correlations between HAM-A score and age, disease duration, and Hoehn & Yahr stage. Linear regression fits are shown by red lines.

**Figure 4:**
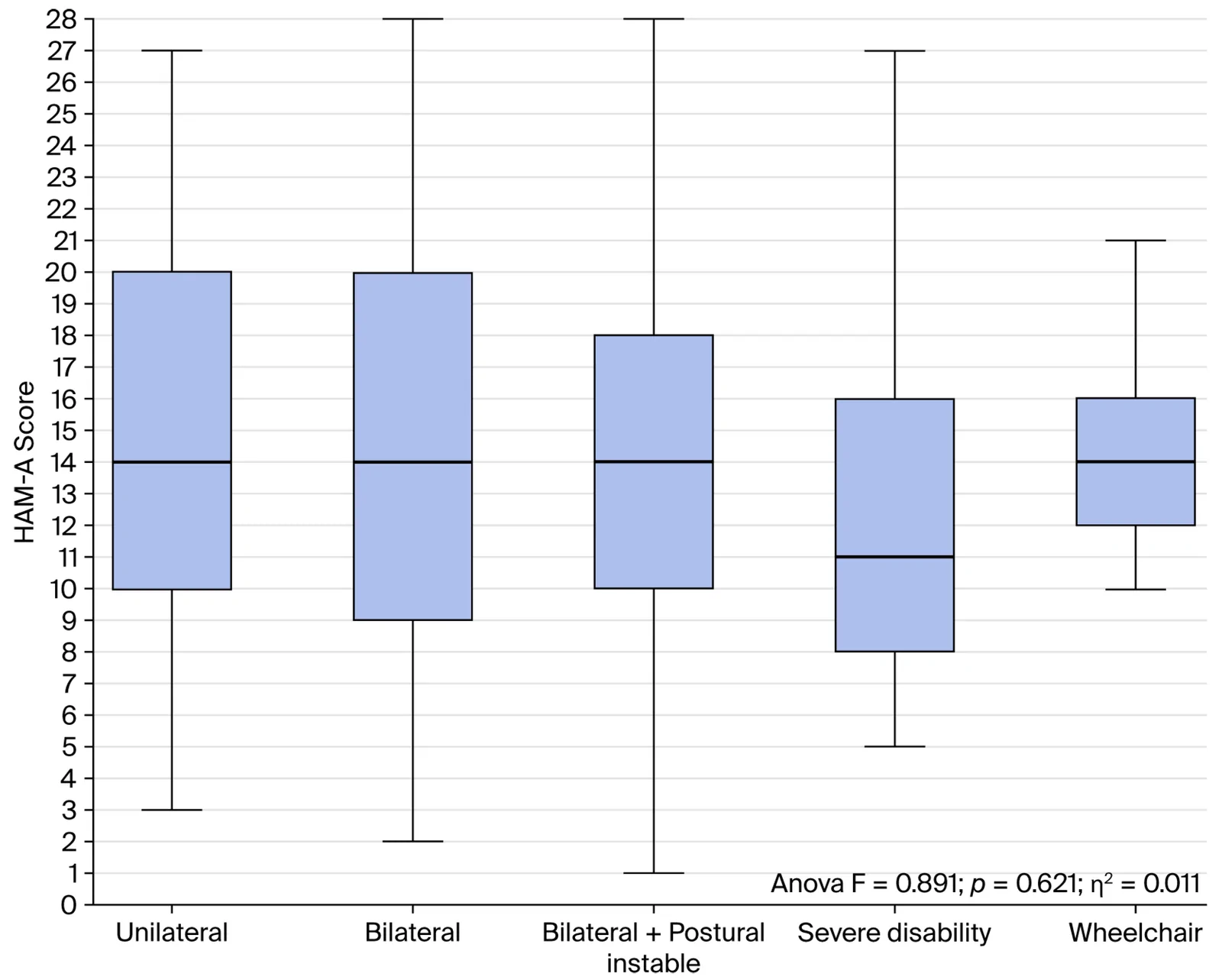
HAM-A scores by Hoehn & Yahr category. ANOVA F = 0.891; *p* = 0.621.

**Figure 5:**
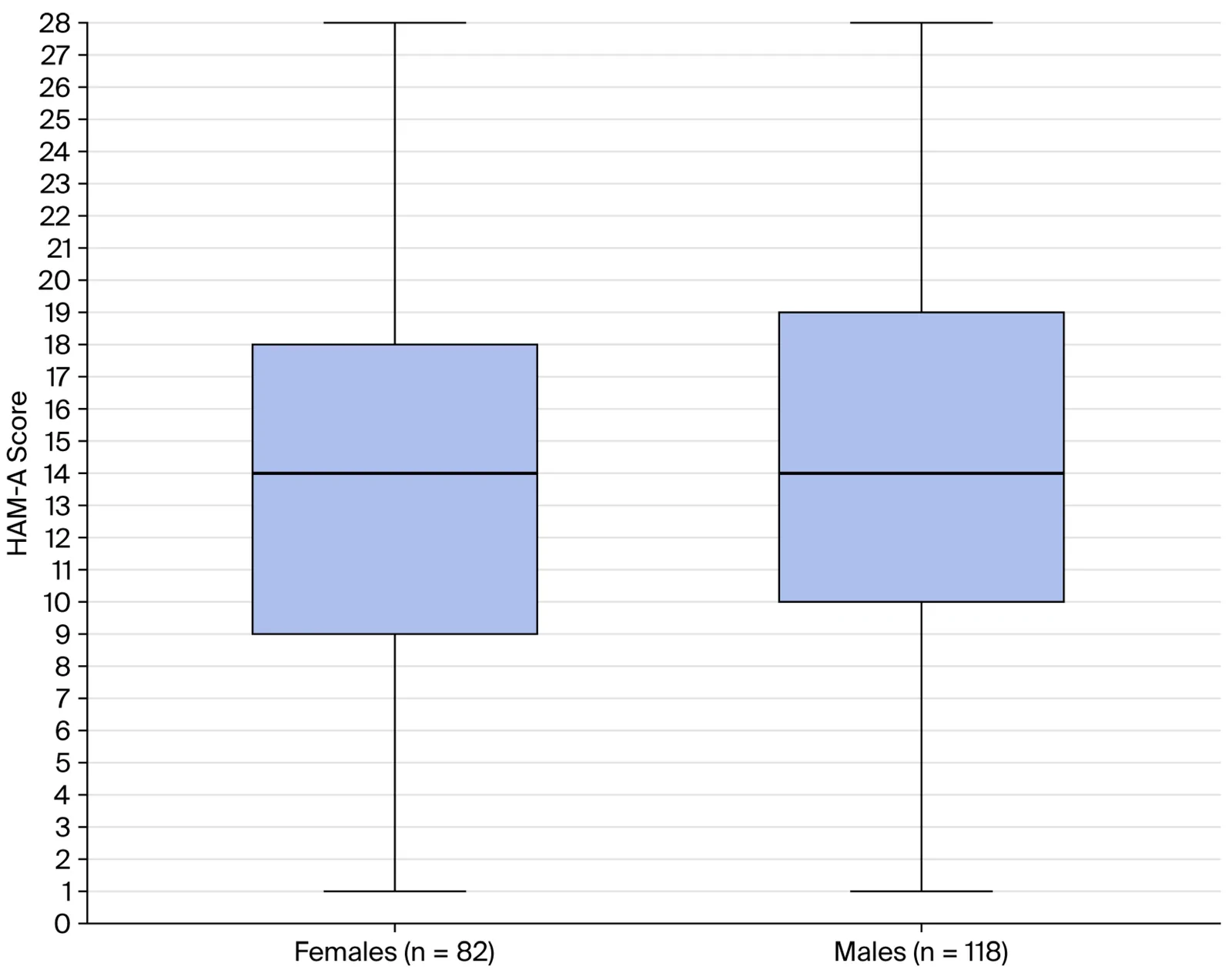
HAM-A scores by sex. t = −0.508; *p* = 0.612.

None of the clinical variables analyzed showed a statistically significant link with the HAM-A score (Table 3, Figure 3). Additionally, a one-way ANOVA conducted across H&Y categories was not significant (F = 0.891; *p* = 0.621; η^2^ = 0.011 Table 4, Figure 4). Pearson’s r was-0.073 (*p* = 0.305) with H&Y stage, 0.115 (*p* = 0.105) with disease duration, and 0.032 (*p* = 0.657) with age. There was no significant difference between the sexes (males: 13.92 ± 6.02 vs. females: 13.76 ± 6.22; t = −0.181; *p* = 0.856; Figure 5).

## 5. Discussion

### 5.1. Prevalence of Anxiety in the Parkinson’s Disease Cohort

Our cohort’s 52.5% HAM-A-detected prevalence of anxiety is consistent with estimates from prior systematic reviews and meta-analyses that show prevalence rates ranging from 25% to 49% across various PD populations [6,15]. Although our figure sits above the upper bound of this range, the discrepancy is not entirely unexpected once methodological and population level factors are taken into account. Anxiety prevalence estimates in PD have historically varied widely depending on the instrument used, the cut-off applied, the stage of disease sampled, and whether the population was drawn from movement-disorder clinics, general neurology services, or community settings. Clinic-based cohorts, such as ours, tend to over-represent patients with more advanced motor and non-motor symptom burden, since these are the patients most likely to be referred for specialist follow-up, and this referral bias plausibly inflates apparent psychiatric morbidity relative to population-based samples.

It is crucial to make clear the underlying premise that guides the interpretation of these results, in addition to determining the general prevalence of anxiety in this group. Only when a significant percentage of patients score higher than the HAM-A threshold does these scores actually represent an anxiety construct separate from the motor and non-motor burden of Parkinson’s disease. If the HAM-A accurately measures anxiety related to Parkinson’s disease, its scores should rise in tandem with indicators of the disease’s severity and progression, such as a longer disease duration or a more advanced motor stage. This would be consistent with the more general clinical finding that psychological distress tends to worsen as motor disability does.

On the other hand, if HAM-A scores continue to be high across the cohort but do not follow these severity indicators, this would imply that the scale may be measuring symptom overlap, such as autonomic dysfunction, tremor, or sleep disturbance, rather than a particular and clinically significant anxiety dimension. In light of this presumption, we next investigated the possibility of a correlation between the clinical severity characteristics gathered in this study and HAM-A scores.

Our adoption of the HAM-A criteria of ≥14 as a cut-off threshold that, as we explore below, may lack specificity in the PD context and may have contributed to the larger estimate in our sample. Cut-off scores developed and validated in primary psychiatric populations are not necessarily transportable to a neurological population in which many of the scale’s constituent items overlap with disease-intrinsic phenomena. When a cut-off calibrated for a population without confounding somatic pathology is applied unmodified to PD patients, the effect is almost mechanically one of inflated apparent caseness: patients who do not meet any reasonable definition of a clinical anxiety disorder can nonetheless cross the threshold purely on the strength of motor and autonomic items. This factor is crucial to the interpretation of our main conclusion and will be mentioned frequently in this discussion.

This high prevalence nevertheless emphasizes the need for regular, structured screening for anxiety disorders as part of comprehensive PD therapy, in line with current global guidelines [16]. The underlying message of these guidelines is still valid even after accounting for the possibility of some degree of overestimation: anxiety in Parkinson’s disease (PD) is common, under-recognized in routine motor-focused consultations, and consequential for quality of life, functional independence, and even motor symptom perception. Therefore, the clinical task is to combine systematic screening with an assessment strategy that can differentiate between true affective pathology and disease-related somatic mimicry, a distinction that a single generic anxiety scale cannot reliably make on its own, rather than dismissing high prevalence figures as artifactual.

### 5.2. Absence of Association with Objective Disease Severity Markers

The primary and therapeutically significant finding of this study is the systematic lack of any significant relationship between HAM-A scores and objective measures of PD severity, such as H&Y stage, illness duration, age, and sex. Alongside motor impairment, increasing reliance on caretakers deterioration, caregiver dependence, and the expectation/possibility of future decline are all credible psychological stressors, it makes sense that impairment anxiety symptoms may be predicted to worsen. However, this is not consistently supported by the literature, and our results show that this pattern of HAM-A scores also does not follow the course of the disease.

Therefore, the lack of any such gradient in our data is a finding that actively undermines the notion that the HAM-A is measuring a construct that fluctuates coherently with the underlying disease process, rather than being a null or uninformative result.

This pattern raises severe concerns about the construct validity of the HAM-A when utilized with PD patients. In line with the larger body of research relating disease burden to psychological distress in chronic neurological illness, at least a slight correlation with H&Y stage or disease duration would be anticipated if the instrument were capturing anxiety as a psychological state separate from motor and autonomic pathology. Instead, our data suggest that a substantial portion of the variance in HAM-A scores is being driven by something structurally independent of clinical disease stage, most plausibly the scale’s own somatic architecture, rather than the psychiatric syndrome it purports to measure. This has direct implications for any research that has used, or plans to use, the HAM-A as an outcome measure in PD treatment trials, since apparent treatment related to changes on the scale could equally well reflect fluctuation in motor or autonomic symptoms rather than genuine anxiolytic effects.

### 5.3. A Mechanistic Explanation: Semiological Overlap Between PD and HAM-A Somatic Items

**Figure 6:**
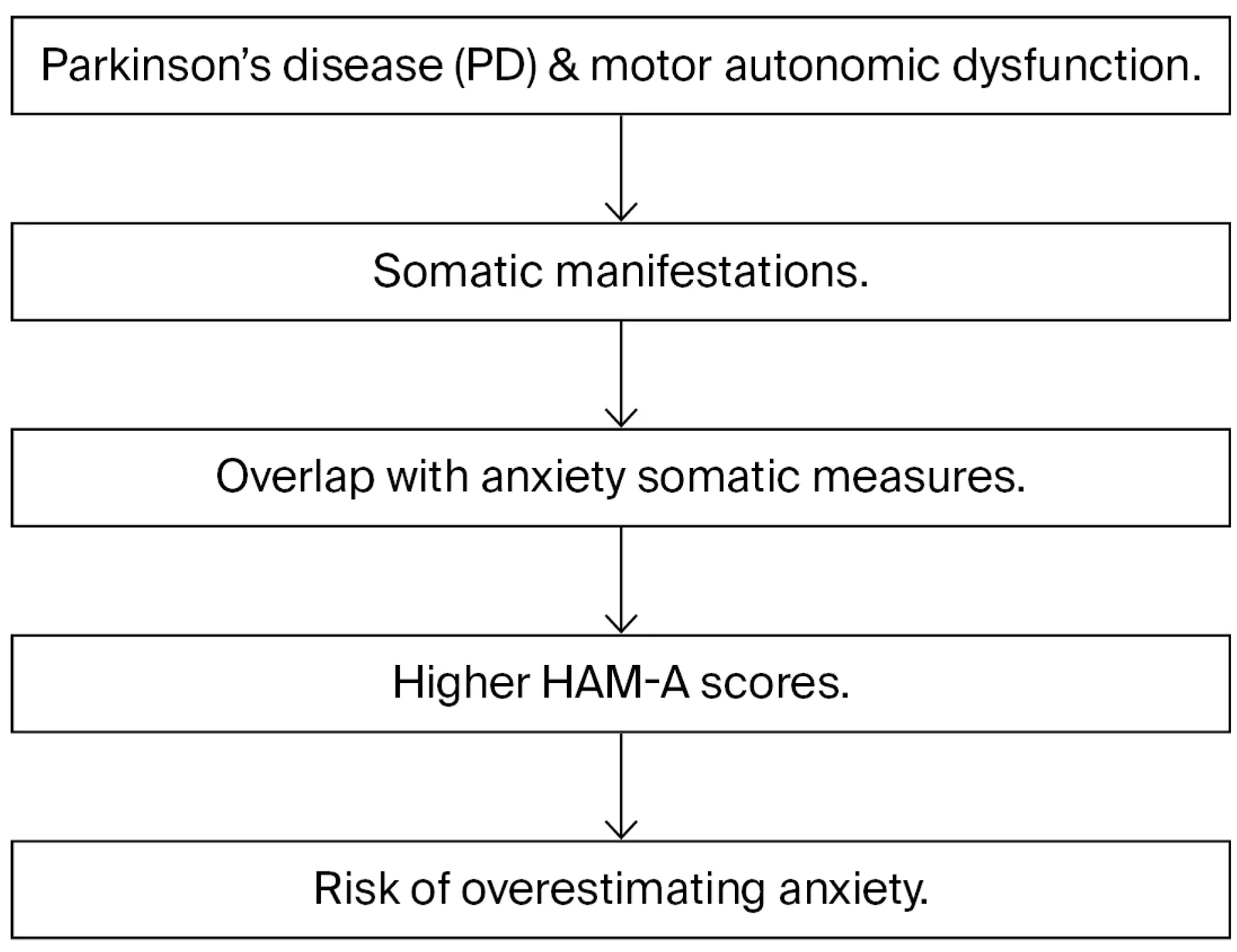
Semiological similarity between HAM-A somatic items and Parkinson’s disease symptoms.

The well-established semiological overlap between the intrinsic motor and autonomic characteristics of Parkinson’s disease (PD) and the somatic subscale items of the HAM-A provides a convincing mechanistic explanation (Figure 6). The HAM-A’s items 7–14 examine autonomic dysfunction, cardiovascular and gastrointestinal symptoms, muscular tension, tremors, and sleep problems. These symptoms can all be obvious signs of Parkinson’s disease (PD), even in the absence of associated anxiety [9,17]. Resting and postural tremor, rigidity-related muscular tension, orthostatic and other autonomic disturbances, gastrointestinal dysmotility, and fragmented, non-restorative sleep are all recognized, disease-intrinsic features of PD that arise from the underlying neurodegenerative process itself, independent of any co-existing mood or anxiety disorder.

Because the HAM-A was designed for, and validated in, populations without a competing organic explanation for these somatic complaints, its scoring algorithm has no mechanism to discount symptoms that are attributable to neurological disease rather than psychiatric illness. Regardless of the source, each endorsed somatic item is only added to the overall score. This implies that the scale is essentially re-measuring the burden of motor and autonomic diseases in PD patients under the guise of worry. This contamination effect, which likely inflates HAM-A levels regardless of illness stage, can account for the lack of expected positive relationships with H&Y stage. Because contamination functions similarly across early, and advanced disease patients at every stage show some degree of tremor, rigidity, or autonomic dysfunction, the somatic inflation is diffused across the entire severity spectrum rather than concentrated at higher H&Y stages, flattening any correlation that might have emerged between total HAM-A score and objective disease severity.

Since the same brainstem and autonomic circuits linked to the neurobiology of anxiety are also linked to the motor and non-motor symptomatology of Parkinson’s disease, this contamination effect is more than just a statistical annoyance. Therefore, either a multimodal assessment approach that triangulates scale scores against clinical interview and contextual information is needed instead of relying solely on somatic symptom counts, or an instrument specifically designed to distinguish between disease-driven autonomic and motor activation and true affective anxiety.

### 5.4. Parkinson’s-Specific Anxiety Phenomenology

These results are consistent with those of Leentjens et al. [9], who systematically demonstrated that some HAM-A somatic items are unable to differentiate between anxiety-related symptoms and PD motor features. The contamination phenomenon mentioned above was empirically confirmed by their research, which used item-level analyses to show that endorsement of somatic anxiety items in PD patients correlated more strongly with motor examination findings than with independent clinical ratings of anxiety severity.

Similarly, Dissanayaka et al. [11] and Broen et al. [6] have highlighted the inadequacy of general anxiety scales in capturing anxiety phenomenology particular to Parkinson’s disease (PD), including fall-related phobias, anticipatory freezing-related anxiety, and OFF-state anxiety. Since none of these occurrences neatly map onto the symptom categories covered by conventional, generic anxiety instruments like the HAM-A; each one deserves independent examination.

In Parkinson’s disease (PD), fall-related phobia is a particular situationally triggered dread that arises in reaction to the patient’s personal experience of postural instability and previous falls. It can become extremely incapacitating, limiting mobility and social involvement considerably beyond what the actual motor disability alone would suggest. It shows up as avoidance of activities, settings, or movements associated with perceived fall risk. Items inquiring about diffuse apprehension, the currency of generic anxiety measures, do a poor job of capturing this dread since it is based on a real, disease-specific risk rather than broad worry.

The worry that arises around the unpredictable beginning of freezing episodes of gait, especially at doorways, turns, or when starting movement under time constraint, is known as anticipatory freezing-related anxiety. Patients frequently describe a self-reinforcing cycle in which anticipatory anxiety about freezing itself causes or exacerbates freezing episodes. This feedback loop between psychological state and motor phenomenon is unique to Parkinson’s disease (PD) and has no equivalent in the symptom architecture of the HAM-A or similar generic instruments.

The term “OFF-state anxiety” describes the noticeable, frequently sudden escalation of feelings of anxiety that many Parkinson’s disease patients encounter during OFF periods, when the dopaminergic medication effect diminishes and motor symptoms resurface. These symptoms usually go away, at least partially, once the ON state resumes.

Because it is time-locked to the motor fluctuation cycle rather than persistent across the day, a single cross-sectional administration of a generic anxiety scale risks either under-detecting it, if given during ON, or misattributing purely fluctuation-related distress to a persistent generalized anxiety disorder, if given during OFF. None of these three phenomena is adequately operationalized by an instrument such as the HAM-A, which was developed for a nosology of anxiety disorders that predates the detailed characterization of PD-specific non-motor fluctuations.

### 5.5. The Confounding Role of Comorbid Depression

It is also important to acknowledge the possible confounding effect of concomitant depression, which affects up to 40% of PD patients and is highly, comorbidly, and significantly associated with anxiety, is a distinct and independent drawback of our study [18]. In Parkinson’s disease (PD), anxiety and depression are not clearly distinct syndromes; rather, they often coexist, share overlapping neural substrates, and exhibit significantly overlapping symptomatology, such as sleep difficulty, weariness, poor attention, and psychomotor shift. In the absence of concurrent depression evaluation, our studies cannot rule out the impact of depressed symptomatology on observed HAM-A scores because the HAM-A contains items with depressing content (item 6).

This limitation compounds the somatic contamination problem discussed above, rather than operating independently of it. A patient with untreated depressive symptoms may endorse HAM-A item 6 on the basis of depressed mood rather than anxious mood, further inflating the total score without any true anxiety disorder being present. Because our design did not include a validated depression measure administered concurrently, we are unable to parse out the variance attributable to depressive symptomatology from that attributable to genuine anxiety, and the possibility that some portion of our reported 52.5% prevalence reflects unrecognized or unmeasured depression cannot be excluded. Future research in this area should, as a matter of methodological principle, administer a validated depression measure alongside any anxiety instrument in PD populations, precisely so that the shared variance between the two constructs can be statistically modeled rather than left as an unexamined source of measurement error.

### 5.6. Clinical and Methodological Implications

Both clinical practice and research methodology are immediately impacted by these findings. When interpreting HAM-A scores in individuals with Parkinson’s disease (PD), clinicians should exercise caution because elevated scores may indicate a motor rather than an emotional issue. It is highly advised to use a multi-source, contextualized assessment method.

Practically speaking, this means that a patient with Parkinson’s disease should never be diagnosed with an anxiety disorder based solely on an elevated HAM-A score, nor should this immediately lead to the start of anxiolytic medication. Rather, a higher score should be interpreted by clinicians as a call for a more thorough clinical evaluation, which should include a review of the patient’s motor state at the time of assessment, a screening for depressive symptomatology, direct questions about situational triggers like fear of freezing or falling, and an explanation of the temporal relationship between reported anxiety and the medication ON/OFF cycle. Whenever possible, confirming information from family members or caregivers can assist differentiation between anxiety that is limited to particular motor states or situational triggers and anxiety that are persistent and widespread.

From the perspective of study technique, our results discourage the careless use of the HAM-A and, consequently, other general anxiety tests created outside of the setting of Parkinson’s disease as the main or exclusive outcome measure in PD anxiety studies. Studies that only use these tools run the risk of overestimating the actual clinical anxiety burden in their samples as well as incorrectly attributing symptomatic change to anxiolytic effects in intervention trials when, in reality, the underlying cause of scale movement may be fluctuations in motor or autonomic symptom control.

This is especially important for studies involving dopaminergic drugs or deep brain stimulation, as improvements in motor and autonomic symptoms may result in an apparent decrease in HAM-A-rated anxiety that is unrelated to any real impact on mood.

### 5.7. Alternative Anxiety Assessment Instruments in Parkinson’s Disease

The particular clinical or research question at hand should be taken into consideration while selecting one of the several highly valid tools for evaluating anxiety related to Parkinson’s disease.

Three clinically significant anxiety characteristics are distinguished by the Parkinson Anxiety Scale (PAS), which was created especially for this population and validated by Leentjens et al. [19]: avoidance behavior, episodic anxiety, and persistent anxiety. Its main advantage over generic instruments is that it was designed with PD-specific phenomenology in mind from the beginning. Rather than depending on a generic somatic symptom checklist that is susceptible to disease contamination, it purposefully included items that capture avoidance of feared situations, discrete episodic anxiety attacks that may include OFF-state anxiety, and more persistent, trait-like anxious symptomatology.

Secondary pilot research using data from a buspirone trial provided early evidence that the PAS is similarly responsive to treatment-related change, with moderate-to-high correlations between changes in PAS scores and changes in clinician- and patient-rated outcome measures [20]. This supports the PAS’s suitability for cross-sectional screening as well as longitudinal and interventional research designs.

A quick, self-reporting instrument that is sensitive enough for outpatient screening is the Generalized Anxiety Disorder-7 (GAD-7) [21]. In busy movement-disorder clinics, where time constraints frequently make lengthy, interviewer-administered instruments impractical, its self-administered structure and conciseness make it appealing for routine clinical usage. However, because it was designed for generalized anxiety disorder in the general medical population, it still has some of the same somatic-overlap vulnerabilities as the HAM-A, though to a lesser extent because it places more emphasis on worry-based and cognitive items than on autonomic and somatic symptoms. Therefore, rather than being used alone as a conclusive diagnostic or outcome measure, it is best used as a first-line screening tool to identify patients for additional assessment.

The Hospital Anxiety and Depression Scale (HADS) offers the useful advantage of measuring anxiety and depression symptoms simultaneously while lowering somatic item contamination [22]. The HADS was created to be useful in populations with concurrent physical illness by purposefully removing somatic items from its anxiety and depression subscales. This makes it a particularly appealing option for use in Parkinson’s disease (PD), as the removal of autonomic and motor symptom items directly addresses the contamination issue found in this study.

Because anxiety and depressive symptoms can be scored and interpreted independently within the same brief administration, its dual-subscale structure also provides a useful solution to the depression-confounding problem mentioned above. This enables clinicians to determine the relative contribution of each syndrome without the need for two distinct instruments.

The gold standard for developing formal diagnostic categories for comprehensive research-grade evaluation is still the Structured Clinical Interview for DSM-5 (SCID-5) [23]. The SCID-5 is a semi-structured diagnostic interview that, in contrast to the self-reported and short clinician-rated scales previously discussed, enables a qualified clinician to determine, symptom by symptom, whether formal DSM-5 diagnostic criteria for a particular anxiety disorder are met. It specifically probes whether reported symptoms are better explained by a general medical condition like Parkinson’s disease.

The SCID-5 is the most rigorous tool currently available for determining “true” psychiatric caseness in PD research because it can formally exclude organically mediated symptoms. However, its administration time and the need for trained raters make it impractical for routine screening or large-scale epidemiological studies; instead, it should only be used in research contexts, complex diagnostic dilemmas, or validation studies of shorter instruments.

When combined, these four tools show a range of trade-offs between diagnostic rigor, PD specificity, conciseness, and the ability to jointly assess depression. The HAM-A’s continued use in PD populations warrants special caution, given the contamination impact seen in the current investigation. No single instrument is ideal for every purpose; instead, instrument selection should be carefully suited to the clinical or research aim.

### 5.8. Broader Implications for Non-Motor Symptom Screening

More broadly, our results support the call made by Titova and Chaudhuri [24,25,26] and others for the systematic integration of validated NMS screening instruments, such as measures related to anxiety, into accepted motor evaluation scales and traditional PD clinical exams. When clinical attention is largely focused on motor assessment, anxiety is just one of several non-motor symptoms that are often overlooked, along with depression, apathy, cognitive impairment, sleep disturbances, and autonomic dysfunction. Our findings provide an additional, more focused justification for this general call: adding an anxiety measure to standard PD diagnostic procedures is insufficient since the instrument selected needs to be resilient to the semiological overlap that this cohort exhibits.

### 5.9. Strengths and Limitations

The use of a widely accepted, internationally validated anxiety rating tool and a well-defined cohort make this study easier to compare to the larger body of research on anxiety prevalence in Parkinson’s disease. Another methodological strength is the systematic investigation of correlations between HAM-A scores and several objective measures of disease severity, including age, sex, illness duration, and H&Y stage. This allowed the contamination hypothesis to be empirically tested rather than merely asserted theoretically.

Nonetheless, several limitations should temper the interpretation of our findings. First, and most importantly, the absence of a concurrent, validated depression measure means that the relative contributions of true anxiety and confounding depressive symptomatology to the observed HAM-A scores cannot be disentangled, as discussed above. Second, the cross-sectional design does not allow us to determine whether HAM-A scores fluctuate with the motor ON/OFF cycle, a question of direct relevance to the OFF-state anxiety phenomenon described by Dissanayaka et al. [11] and Broen et al. [6]. Third, our reliance on a single generic instrument, rather than a PD-specific tool such as the PAS or a diagnostic interview such as the SCID-5, means that we are only able to demonstrate contamination indirectly, through the absence of expected correlations, rather than directly through item-level or convergent-validity analyses against a PD-specific comparator. Finally, as a clinic-based sample, our cohort may not be representative of the full spectrum of PD severity and non-motor burden encountered in community populations, which may limit the generalizability of the prevalence estimate reported here, though it does not undermine the internal validity of the construct-validity concerns raised by our correlational findings.

### 5.10. Future Research Directions

Building on the limitations outlined above, several directions for future research follow naturally from our findings. Prospective studies that administer the HAM-A, alongside a PD-specific instrument such as the PAS, and a validated depression scale, would allow direct quantification of the degree of somatic-item contamination and its interaction with comorbid depressive symptomatology. Studies that assess anxiety symptoms separately during defined ON and OFF motor states would help clarify the extent to which OFF-state anxiety, rather than a stable trait-like anxiety disorder, accounts for elevated scores in fluctuating patients. Longitudinal designs tracking HAM-A, PAS, and HADS scores across disease progression would also help establish whether the flat relationship we observed between HAM-A scores and H&Y stage reflects a genuine absence of association between anxiety and disease severity or is itself an artefact of the somatic contamination effect masking a true underlying relationship. Finally, validation studies directly comparing HAM-A-derived caseness against SCID-5 diagnoses in PD samples would provide the most definitive test of the construct-validity concerns raised here, and could inform the derivation of a PD-adjusted HAM-A cut-off or a modified, somatic-item-reduced scoring algorithm analogous to the approach already taken by the HADS.

The significant semiological overlap between the motor and dysautonomic symptoms of Parkinson’s disease (PD) and the somatic items of the Hamilton Anxiety Rating Scale (HAM-A) is depicted in Figure 6. When evaluated using non-specific measures like the HAM-A, a number of PD cardinal symptoms, including resting tremor, axial rigidity, hypersalivation, bowel problems, hyperhidrosis, and orthostatic hypotension, may be incorrectly graded as worrying features.

## 6. Conclusions

Overall, the present findings indicate that a high HAM-A-detected anxiety prevalence in PD should be interpreted with considerable caution, given the well-documented semiological overlap between the scale’s somatic items and the intrinsic motor and autonomic features of the disease. The absence of any meaningful correlation between HAM-A scores and objective markers of disease severity, together with the compounding influence of unmeasured depressive symptomatology, argues strongly against continued reliance on generic, psychiatrically derived anxiety instruments in this population. A contextualized, multi-source assessment strategy incorporating PD-specific tools such as the PAS, somatic-item-reduced instruments such as the HADS, and, where feasible, structured diagnostic interviews such as the SCID-5 alongside routine, guideline concordant screening, represents the most defensible path toward accurately characterizing and ultimately treating anxiety in Parkinson’s disease.

## Figures and Tables

**Table 1:** The research population’s clinical and demographic features (*n*= 200).

Variable	Value (Mean ± SD)	Range/*n* (%)
200 patients in total	200	—
Male/Female	118/82	59%/41%
Average age (years)	62.77 ± 8.32	[43.7–83.8]
Duration of disease (years)	3.42 ± 1.76	[0.5–9.3]
Average HY stage	2.59 ± 0.91	[1.0–5.0]
Average HAM-A score	13.85 ± 6.09	[0–28]
HAM-A ≥ 5 (anxious)	186/200	93.0%
HAM-A ≥ 15 (moderate+))	89/200	44.5%
HAM-A ≥ 22 (severe)	19/200	9.5%

**Table 2:** Statistical analysis: HAM-A score-related factors.

Variable	HAM-A Score	Statistic	*p*-Value
Female (*n* = 82)	13.76 ± 6.22	t = −0.181	0.856 (NS)
Male (*n* = 118)	13.92 ± 6.02		
Age—Pearson r	r = 0.032	—	0.657 (NS)
Disease duration—Pearson r	r = 0.115	—	0.105 (NS)
HY stage—Pearson r	r = −0.073	—	0.305 (NS)
HY stage—ANOVA	—	F = 0.891	0.621 (NS)

NS: not significant.

**Table 3:** Distribution of patients by HAM-A diagnostic category.

Anxiety Category	HAM-A Score	Number of Patients (*n*)	Percentage (%)
No anxiety	<14	95	47.50%
Moderate anxiety	14–17	46	23.00%
Severe anxiety	≥18	59	29.50%

**Table 4:** Group comparisons and Pearson correlations between clinical factors and the HAM-A score.

Variable	Statistic	*p*-Value	Significant
Age	r = 0.032	0.657	No
Disease duration	r = 0.115	0.105	No
Hoehn & Yahr stage	r = −0.073	0.305	No
Sex (*t*-test)	t = −0.181	0.856	No
HY category (ANOVA)	F = 0.891	0.621	No

## Data Availability

The datasets generated during this study are available from the corresponding author upon reasonable request.
